# Editorial: Burnout syndrome and PTSD in physicians and their relation after COVID-19

**DOI:** 10.3389/fpsyg.2024.1358020

**Published:** 2024-01-22

**Authors:** Carlos Aníbal Martínez-Martínez

**Affiliations:** National Autonomous University of Honduras, Tegucigalpa, Honduras

**Keywords:** COVID-19, burnout, depression, anxiety, physicians, nurses

There was a rise in stress levels, depressive symptoms, and anxiety in the general population during the COVID-19 pandemic. This was something not just limited to the risk of getting infected but also post-infection mental health consequences, ranging from moderate to severe anxiety and depressive symptoms that could last even 30 months post-exposure to SARS-CoV2 (Hummel et al., 2021). Medical professionals experienced a higher burden while working at a faster pace, having less time to process trauma, and they were also prone to developing post-traumatic stress symptoms and post-traumatic stress disorder (Riedel et al., 2021). Moreover, healthcare professionals were affected by external factors such as institutional limited capacities, being surrounded by quarantined individuals, disrupted social support during isolation, and lack of personal protective equipment, all creating distress among frontline health workers during the COVID-19 pandemic (Sultana et al., 2020). In addition, while this was identified, there were other healthcare professionals developing techniques or taking advantage of the digital era to treat and prevent mental illnesses in medical professionals. Such is the case for online psychological assistance services that were widely used during the COVID-19 pandemic (Dincer and Inangil, 2021).

This Research Topic collects four research articles that describe different geographic populations of frontline healthcare professionals during the pandemic, and the psychosocial effects of taking care of patients, working with blurry guidelines, extensive working hours, and the protective factors that prevent developing burnout, depression, and anxiety symptomatology.

Maghsoodi et al. conducted a qualitative study on nurses, with the goal of determining their perceptions of work and life during the COVID-19 pandemic. They took an approach using interviews, which provided different and individualized perspectives of the matter. However, their results converged into similar concerns, such as team cooperation and communication challenges in difficult work conditions, or suffering and dissatisfaction with unfair wages and benefits. The authors divided the findings into main themes that were classified as an unsafe work environment and the shadow of suffering and death. This study contributed to showing the perspectives and sufferings of healthcare professionals that have most contact with the patient, and the psychological, professional, and social toll the pandemic had on them. On the other hand, in Italian physicians and nurses, Epifanio et al. explored the existence of a positive correlation between hopelessness and burnout, and the role of Trait Emotional Intelligence in this relationship. They concluded that there were significant burnout level differences in genders, professional profiles, and working zones, but also that Trait Emotional Intelligence presented a protective role on healthcare workers' mental health. In a similar vein Hu et al. conducted a study in China and analyzed the different psychological states of medical professionals during the COVID-19 pandemic to provide a reference for the formulation of a psychological crisis intervention plan. Their results showed that female workers were more prone to the negative effects of depression and hypochondriac emotional stress. However, doctors and nurses were all affected by depression, neurasthenia, obsessive anxiety, and stress. Based on these results, they recommended having psychological counseling for their front-line staff and to improve their emergency management practices. Another study in China was conducted by Yin et al. evaluating the effects of long working hours and depressive symptoms present in medical staff. They concluded that most medical professionals that worked more than 8 h per day presented depressive symptoms. Factors like family support or organizational support showed relevance when mitigating burnout and depressive symptoms among medical professionals in this study. These findings can help to develop structures that allow medical professionals to have a better work-life balance and to avoid the deleterious effects of being overworked, especially in situations like the COVID-19 pandemic.

In view of the findings of this Research Topic, identifying the risk and protective factors of mental health issues among physicians and nurses should be a priority in emergencies situations, not forgetting that they are humans and the role they play in high stress and high responsibility situations might have a deleterious psychosocial effect on them. Psychological support structures should be developed to identify, treat, and prevent further psychological damage in front-line healthcare providers in situations of emergencies such as the COVID-19 pandemic.

## Author contributions

CM-M: Writing—original draft, Writing—review & editing.

## References

[B1] Dincer B. Inangil D. (2021). The effect of emotional freedom techniques on nurses' stress, anxiety, and burnout levels during the COVID-19 pandemic: a randomized controlled trial. Explore 17, 109–114. 10.1016/j.explore.2020.11.012 33293201 PMC7834511

[B2] Hummel S. Oetjen N. Du J. Posenato E. de Resende Almeida R. M. Losada R.. (2021). Mental health among medical professionals during the COVID-19 pandemic in eight european countries: cross-sectional survey study. J. Med. Int. Res. 23, e24983. 10.2196/24983 33411670 PMC7817254

[B3] Riedel B. Horen S. R. Reynolds A. Hamidian Jahromi A. (2021). Mental health disorders in nurses during the COVID-19 pandemic: implications and coping strategies. Front. Pub. Health 9, 707358. 10.3389/fpubh.2021.707358 34765579 PMC8575697

[B4] Sultana A. Sharma R. Hossain M. M. Bhattacharya S. Purohit N. (2020). Burnout among healthcare providers during COVID-19: challenges and evidence-based interventions. Indian J. Med. Ethics V, 1–6. 10.20529/IJME.2020.73 34018959

